# Ultra-high temperature bacterial agents enhance heavy metal passivation and antibiotic degradation in compost

**DOI:** 10.3389/fmicb.2025.1708982

**Published:** 2025-11-25

**Authors:** Zifang Chi, Shengyi Zhang, Yuru Li, Huai Li

**Affiliations:** 1 Key Lab of Groundwater Resources and Environment, Ministry of Education, Jilin University, Changchun, China; 2 Northeast Institute of Geography and Agroecology, Chinese Academy of Sciences, Changchun, China

**Keywords:** bioaugmentation, co-contamination, synergistic mechanism, livestock manure, resource recovery

## Abstract

The co-contamination of heavy metals and antibiotics in livestock manure presents a significant environmental challenge. This study developed an integrated ultra-high-temperature (UHT) composting process to address this issue. Through an L_9_(3^4^) orthogonal experiment, we optimized key parameters and identified 60% moisture content, 25:1 carbon-to-nitrogen (C/N) ratio, and 10% (w/w) activated carbon addition as the optimal combination, with activated carbon being the most influential factor. In pilot-scale validation, bioaugmentation with a specialized UHT microbial consortium (UHTMC) at 1.0% dosage achieved a peak temperature of 84.3 °C, maintaining temperatures above 70 °C for 15 days and above 50 °C for 28 days, which significantly surpassed the control. This enhanced thermophilic profile drove superior co-removal performance: 56.0% copper (Cu) and 57.3% zinc (Zn) passivation rates, representing improvements of 11.3 and 11.1 percentage points over the control, respectively, and near-complete degradation (> 99.4%) of tetracycline (TC), oxytetracycline (OTC), and chlortetracycline (CTC). This work demonstrates that bioaugmented UHT composting synergistically enhances microbial activity, effectively passivates heavy metals, and completely degrades antibiotics, providing an efficient resource-oriented strategy for treating co-contaminated solid waste.

## 1 Introduction

With the rapid advancement of global urbanization and economic development, the annual production of organic solid waste continues to surge. This posing severe challenges for ecosystem security and human health. In China, livestock manure output reaches 3.8 billion tons annually. Compostable components (e.g., food waste) account for 30–50% of municipal household waste (Liu et al., 2024; Li et al., 2015). Improper disposal of such waste generates leachate, which contaminates groundwater and releases potent greenhouse gases. For example N_2_O, which has a single-molecule warming potential 298 times that of CO_2_. Traditional landfilling occupies vast land resources and leads to methane emissions under anaerobic conditions (contributing to 16% of global anthropogenic emissions) (IPCC, 2021). Incineration poses risk of releasing toxic substances such as dioxins (Huang, 2022). In contrast, composting technology has synergistic advantages, providing reductiong, harmlessness, and resource recovery, which converts organic matter into stable humus through microbial metabolism. This process produces high-quality organic fertilizer and aligning with circular economy principles (Bernal et al., 2009; Ho et al., 2022).

Composting efficiency and product quality are regulated by key parameters: temperature must be maintained within the thermophilic range (50–65 °C) to maximize organic matter degradation and eliminate over 90% of pathogenic bacteria (Liang et al., 2003); moisture content between 40% and 60% optimally supports microbial metabolism. Deviations from this range can lead to anaerobic conditions (e.g., hydrogen sulfide production), which may subsequently inhibit enzyme activity (Liang et al., 2003; Ghanney et al., 2023). And a carbon-to-nitrogen (C/N) ratio of 25:1–35:1 balances microbial nutrient demand, preventing nitrogen loss or delayed maturity (Shen et al., 2024). The microbial community structure, particularly the dominance of *Firmicutes* and *Actinobacteria* (> 80% during thermophilic phases), directly determines the degradation efficiency of cellulose and hemicellulose (Shen et al., 2024; Yu et al., 2018; Sánchez-Vázquez et al., 2018). Recent advances include exogenous additives such as biochar, which utilizes its high specific surface area to adsorb heavy metals and improve porosity, shortening composting cycles by approximately 20% (Zhou et al., 2018; Nguyen et al., 2022), and ultra-high-temperature microbial consortia (e.g., *Thermus* and *Geobacillus* strains) that elevate temperatures above 80 °C for 5–7 days, significantly enhancing antibiotic removal (e.g., tetracycline degradation rates up to 95%) and reducing antibiotic resistance genes (ARGs) (Liao et al., 2018; Cui et al., 2019; Yu and Zhou, 2020).

Despite these promising developments, critical challenges remain unresolved. Firstly, the protracted duration of traditional composting (45–60 days) limits its feasibility for large-scale applications (Xiao et al., 2009). More fundamentally, while individual additives or inoculants show efficacy, the synergistic mechanisms underlying their combined application-particularly for the co-remediation of heavy metals and antibiotics-are poorly elucidated and seldom quantified (Guan et al., 2025). For instance, biochar effectively passivates heavy metals like Cu and Zn through adsorption (reducing their bioavailable fractions by 20–30%), yet it exhibits limited capacity for direct antibiotic degradation (e.g., tetracycline degradation rates often remain below 40%), relying instead on sequestration that may entail long-term environmental risks (Nguyen et al., 2022). Conversely, microbial degradation processes, though highly efficient in breaking down organic pollutants (with degradation rates exceeding 90%), are frequently undermined by the biocidal effects of co-existing heavy metals; studies indicate that bioavailable Cu or Zn at concentrations as low as 50 mg/kg can suppress critical enzymatic activities by over 60% (Harms et al., 2011). This mechanistic ambiguity-the inability to discern whether these interactions are merely additive or truly synergistic-impedes the rational design of integrated processes and reduces optimization efforts to empirical guesswork.

The emerging ‘all-in-one' remediation strategy, which targets the synergistic removal of multiple pollutants within a unified system, represents a frontier in environmental technology and offers a compelling framework to overcome these limitations (Li et al., 2025). Inspired by this paradigm, we hypothesize that a highly efficient composting process for co-contaminated waste can be developed through the integrated and sequential optimization of physicochemical parameters and bioaugmentation. The primary scientific novelty of this work lies in a two-stage experimental strategy, explicitly designed to deconvolute the complex interactions within the composting system and directly address the forementioned mechanistic “black box.”

In the first stage, an L_9_(3^4^) orthogonal design was used to determine the optimal combination of key parameters (moisture content, C/N, and activated carbon dosage), thereby establishing a robust, scientifically-grounded baseline for co-contamination remediation. Subsequently, we assessed the dose-dependent effects of a specialized UHTMC within this optimized physicochemical framework. This structured approach enables a clear discrimination between the contributions of parameter optimization and bioaugmentation, thereby facilitating a direct investigation of their synergy and underlying mechanistic pathways.

In summary, this study offers both a validated, efficient process with clear-cut operational parameters for treating pig manure-straw mixtures, and a methodological framework to decipher synergistic mechanisms in complex bioremediation systems.

## 2 Materials and methods

### 2.1 Experimental materials

#### 2.1.1 Composting raw materials and conditioners

Fresh pig manure and soybean straw used in the experiment were collected from a livestock farm in Sichuan and an agricultural base in Shandong, respectively. After natural air drying, crushing, and screening (2 mm), pig manure and straw were mixed evenly. The basic physicochemical properties are listed in Table 1. Commercially powdered activated carbon (mesh size: 200 mesh, specific surface area: 800–1,200 m^2^/g) was used in the experiment.

**Table 1:** Basic physicochemical properties of composting materials.

**Indicator**	**Water content (%)**	**TOC (g/kg)**	**TN (g/kg)**	**C/N**	**Cu (mg/kg)**	**Zn (mg/kg)**
Pig manure	78.62	386.63	12.58	14.02	105.62	324.36
Soybean straw	5.23	430.21	5.59	72.88	15.36	25.66

To simulate typical composite pollution conditions during composting, the experiment began by uniformly spraying methanol standard solutions of the target antibiotics (TC, CTC, and OTC) onto the mixed material through exogenous addition, ensuring that their initial concentrations reached 10 mg/kg (on a dry weight basis). After addition, the mixture was left undisturbed for 24 h to allow the solvent to fully evaporate and achieve initial equilibrium.

#### 2.1.2 Ultra-high temperature microbial inoculum

The UHTMC used in this study was a commercial product (Shuoyuan Ecological Agriculture Co. Ltd., China). According to the manufacturer's certificate, the defined UHTMC comprises three key thermophilic strains with a total viable count of ≥ 5.0 × 10^9^ CFU/g and an approximate composition of: *Geobacillus stearothermophilus* (≈ 40%, strain GZ-1), *Saccharomonospora viridis* (≈ 35%, strain SV-3), and *Thermomyces lanuginosus* (≈ 25%, strain TL-2). These strains were selected for their functional complementarity in UHT composting: *G. stearothermophilusand T. lanuginosussynergistically* degrade lignocellulose through the production of thermostable enzymes and cellulases, respectively, while *S. viridis*c ontributes to humification and heavy metal biosorption. Prior to use, the inoculum was activated in sterile sucrose solution (5% w/v, 1:50 ratio) on a shaking table at 55 °C for 1 h.

### 2.2 Experimental design

This study was conducted in two stages: optimizing basic parameters via a small-scale orthogonal experiment, followed by validating the dose effect of the UHT inoculum at pilot scale.

#### 2.2.1 Orthogonal optimization experiment for composting pilot test (Phase I)

A three-factor, three-level orthogonal experimental design L_9_(3^4^) was adopted to optimize key composting parameters: moisture content (55%, 60%, 65%), activated carbon addition (0%, 5%, 10%), and carbon-to-nitrogen (C/N) ratio (20:1, 25:1, 30:1). The nine specific parameter combinations (Table 2) were achieved using urea and glucose for C/N adjustment. The composting process was simulated in 2L plastic buckets placed in a constant-temperature blast incubator. Each bucket was filled to 3/4 height with thoroughly mixed materials and sealed with a perforated lid to maintain minimal air permeability. Aerobic conditions were sustained through manual shaking, thereby facilitating the investigation of parameter influences under a controlled temperature regime.

**Table 2:** Level design of orthogonal experimental factors and material ratio.

**Number**	**Water content (%)**	**Activated charcoal (%)**	**C/N**	**Pig manure (g)**	**Soybean straw (g)**
ED1	55	0	20	600	300
ED2	55	5	30	600	300
ED3	55	10	25	600	300
ED4	60	0	30	600	300
ED5	60	5	25	600	300
ED6	60	10	20	600	300
ED7	65	0	25	600	300
ED8	65	5	20	600	300
ED9	65	10	30	600	300

The temperature setting of the incubator simulates the temperature variation curve of standard aerobic composting; the initial 2 days are a temperature rise period (from 25 °C to 55 °C), followed by a high temperature period of maintaining above 55 °C for 5 days, and finally entering a cooling period (slowly decreasing to ambient temperature). Each barrel was weighed daily and sterile water was added to maintain the preset moisture content. To ensure an aerobic environment, the barrel was shaken manually body for 1–2 min daily.

#### 2.2.2 Dose-response experiment of pilot-scale compost inoculum (Phase II)

Based on the optimal parameter combination obtained from the orthogonal experiment, verification and dose-response experiments were conducted in a 70 L forced-draft composting reactor. Three treatment groups were established: control group (CK, 0% inoculum), low-dose group (CT1, 0.5% inoculum), and high-dose group (CT2, 1.0% inoculum).

The mixed materials prepared according to the optimized parameters were uniformly loaded into the reactor. To ensure reproducibility and rigorously monitor spatial temperature uniformity, a comprehensive 5-point thermocouple array was strategically deployed within the reactor. The sensors were positioned at the following critical locations: the geometric center (C) of the pile, the upper layer (U, 10 cm below the top surface), the lower layer (L, 10 cm above the base), and two radial points (R1 and R2) at mid-height, 5 cm from the internal reactor wall, and diametrically opposite to each other. Temperature data from all points were recorded automatically at 2-h intervals throughout the 30-day composting period. The results demonstrated a high degree of thermal homogeneity, with a maximum temperature variation of ≤ 3.0 °C observed across all monitoring points.

An intermittent forced-draft aeration mode was adopted, with an aeration rate controlled at 0.4 L·min^−1^·kg^−1^ of organic matter (15 min on/45 min off). Manual turning of the compost was performed every 3 days to ensure aerobic conditions and promote uniform moisture evaporation. Throughout the process, the composting temperature was entirely driven by microbial metabolic heat without any external heating, while the external environment was maintained at the natural ambient temperature.

### 2.3 Sampling and analysis

The sampling scheme for both stages was consistent. Samples were collected on days 0, 3, 7, 12, 20, and 30 of composting. During each sampling, approximately 50 g of sample was taken from different locations (upper, middle, and lower) in each small-scale test barrel or pilot-scale compost pile. After mixing, the samples were divided using a quartering method. One portion of the fresh sample was immediately used to measure pH, electrical conductivity (EC), moisture content, and germination index (GI); the other portion was freeze-dried, ground, and sieved, then stored at −20 °C for subsequent determination of total organic carbon (TOC), total nitrogen (TN), heavy metal forms, and antibiotic residues.

pH and EC were measured by potentiometry after extraction with a water-soil ratio of 10:1 (w/v); moisture content was determined using a 105 °C oven drying method; TOC was digested with concentrated H_2_SO_4_ and 0.8 mol/L K_2_Cr_2_O_7_, and the organic matter and TN of compost were measured using an external heating-potassium dichromate oxidation method; The GI was determined using radish seeds (Harms et al., 2011).

The total amounts of Cu and Zn were determined using a flame atomic absorption spectrophotometer after digestion on a strong-acid hot plate. Morphological analysis was conducted using an improved BCR sequential extraction method that separates heavy metals into acid-extractable, reducible, oxidizable, and residual states (Guo et al., 2012). Antibiotic residues in compost samples were determined using high-performance liquid chromatography (HPLC) (Nada, 2015).

Data collection and preliminary calculations were performed in Microsoft Excel 2021. Statistical analysis was conducted using the SPSS software (version 26.0). Range analysis and analysis of variance (ANOVA) were performed on the results of the orthogonal experiment to determine the significance and optimal combination of each factor (significance level *p* < 0.05). One-way ANOVA was performed on the indicators between the different treatment groups. All charts were drawn using Origin 2022 software.

## 3 Results and discussion

### 3.1 Optimizing composting parameters through orthogonal experiments to improve pollutant removal

#### 3.1.1 Changes in basic physicochemical parameters of compost

Physicochemical parameters (pH, EC, moisture content, and organic matter) are core indicators of the metabolic activity and decomposition of composting microorganisms. These dynamic changes directly reflect the regulatory effects of various factors on the composting microenvironment. The key characteristics of the changes in each group are as follows:

**(1) Dynamic changes in pH**

**Figure 1:**
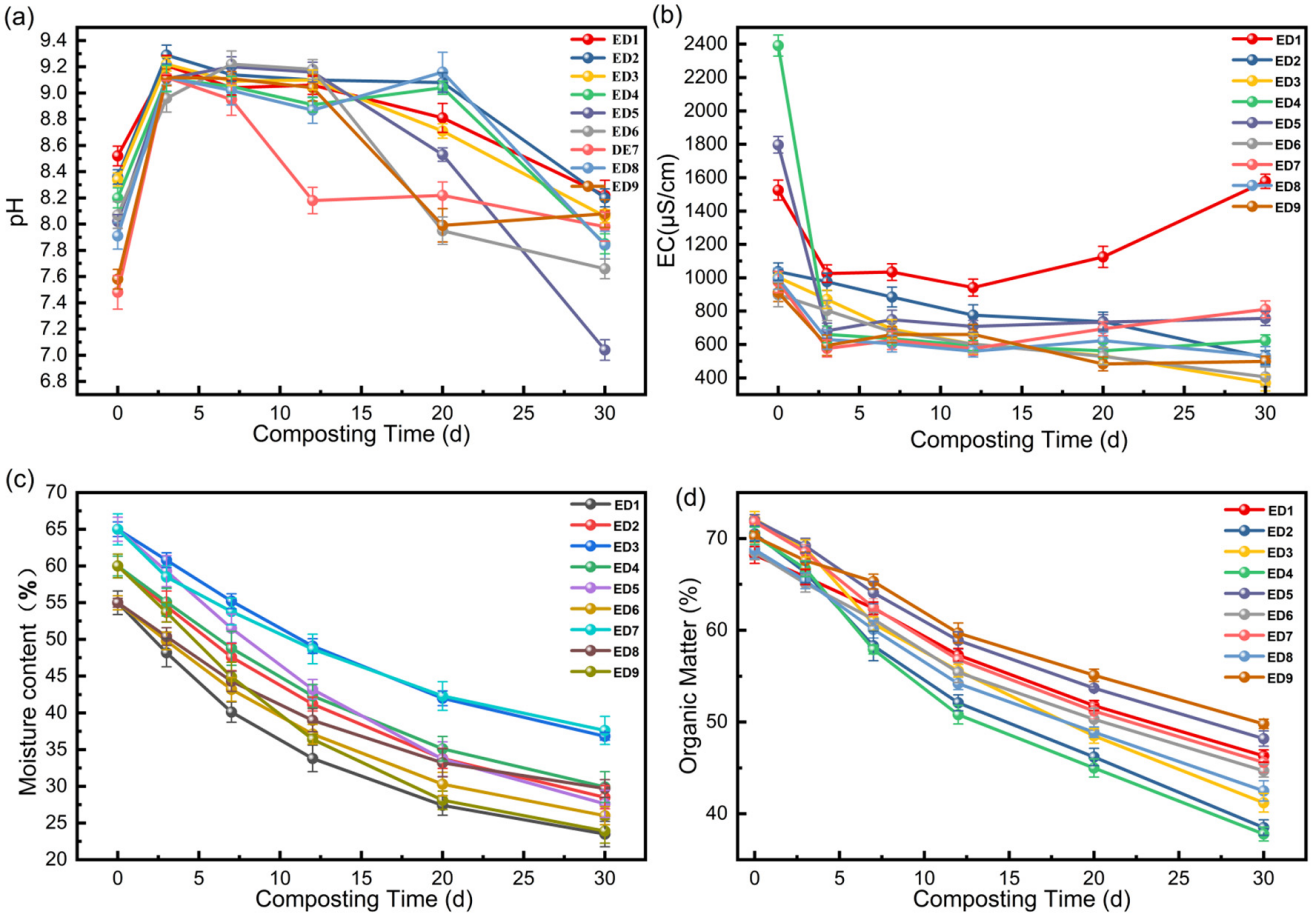
Changes in physicochemical properties of each treatment group (ED1-ED9) in orthogonal experiment: **(a)** pH; **(b)** EC; **(c)** Moisture content; **(d)** Organic Matter.

As shown in Figure 1a, the pH values of all treatment groups exhibited a typical composting characteristic of “an initial increase followed by a decrease”, which is consistent with the acid-base balance law of compost maturity (Rauret et al., 1999). In the initial stage (day 0), the pH values of each group ranged from 7.48 to 8.52, with differences arising from the basic properties of the raw materials and the initial adsorption effect of activated carbon. On day 3, due to the rapid mineralization of organic nitrogen into ammonia, the pH values of all groups reached their peak (8.96-9.29), with ED2 (60% moisture content, 25:1 C/N, 5% activated carbon) having the highest peak value (9.29), reflecting the enhancement effect of carbon-nitrogen balance on ammonification efficiency. By the end of composting on day 30, the pH values of all groups stabilized at 7.04–8.22 [within the range of 7.0–8.5 as per the “Organic Fertilizer” standard (GB/T 25246-2010)]. ED5 (65% moisture content, 25:1 C/N, 0% activated carbon) showed the largest decrease (from 8.02 to 7.04), as a high moisture content significantly affected the degradation and humification of lignocellulose in the compost pile, promoting the accumulation of organic acids (Ghanney et al., 2023). Conversely, ED1 (55% moisture content, 20:1 C/N, 0% activated carbon) showed the smallest decrease (from 8.52 to 8.22), as low moisture content inhibits the acid-producing efficiency of microorganisms (Ghanney et al., 2023). This trend is consistent with the findings of Shen et al., who described the regulation of pH by nitrogen transformation during composting (Guo et al., 2012). Specifically, initial ammonification raises the pH, while subsequent nitrification, which consumes ammoniacal nitrogen and produces nitrate nitrogen, leads to a pH decrease.

**(2) Changes in EC**

The EC value reflects the concentration of soluble ions, and the changes in each group reflect the combined effects of material transformation and microbial ion absorption (Figure 1b). In the initial stage (day 0), there were significant differences in EC values (899–2,391 μS/cm). ED4 (60% moisture content, 25:1 C/N, 10% activated carbon) had the highest EC value because of the high initial salt content of the raw material, while ED6 (55% moisture content, 25:1 C/N, 5% activated carbon) had the lowest EC value because of activated carbon adsorption. During the composting process, the EC values of most groups decreased in the early stage (day 0–7) due to microbial fixation of salt and showed differentiation in the middle and later stages. ED1 (without activated carbon) rebounded to 1,578 μS/cm due to greater ion release than absorption, while ED3 (10% activated carbon) decreased to 370 μS/cm (the lowest value) due to continuous adsorption. Ultimately, ED3 showed the best salt control effect, confirming the fixing effect of activated carbon on soluble ions, which is consistent with the results of Wang et al. (2022).

**(3) Changes in moisture content**

The moisture content of each treatment group continuously decreased with composting, reflecting the combined effects of microbial metabolic consumption and water evaporation (Figure 1c). Initially, it was maintained at 55–65% as designed, decreasing rapidly in the first 15 days (microbial heat production accelerates evaporation) and slowing down in the later stages. By day 30, ED1 (initial moisture content of 55%) had the lowest moisture content (23.5%), whereas ED7 (initial moisture content of 65%) had the highest (37.6%). The final moisture content of the 55–60% initial moisture content group (23.5–29.9%) fell within the ideal range for decomposition (20–30%), while that of the 65% group (36.8–37.6%) was slightly higher, which may lead to local anaerobiosis. This is consistent with the conclusion reported by Wang et al. (2022) and Shen et al. (2024) that the “optimal initial moisture content is 55–60%”.

**(4) Changes in organic matter**

Organic matter degradation reflected microbial metabolic efficiency, and all groups exhibited a continuous downward trend (Figure 1d). In the initial stage, the content increased with an increase in C/N (68.2–72.1%). After 30 d of composting, ED4 (60% moisture content, 25:1 C/N, 10% activated carbon) showed the most thorough degradation (decreased to 37.8%, with a degradation rate of 46.2%), whereas ED9 (60% moisture content, 30:1 C/N, 0% activated carbon) had the highest residual content (49.8%, with a degradation rate of 29.1%). The C/N = 25:1 group exhibited the best organic matter degradation efficiency due to the carbon-nitrogen balance, which was suitable for microbial metabolism, coupled with the enrichment function of activated carbon, consistent with Zhang et al.'s conclusion that “appropriate C/N and activated carbon synergistically enhance degradation rate” (Wang et al., 2022).

#### 3.1.2 The impact of orthogonal experiments on heavy metal passivation

The bioavailability of heavy metals (Cu, Zn) depends on their speciation distribution (exchangeable > reducible > oxidizable > residual). During the composting process, all groups undergo a “transformation from highly active to stable states”. The key characteristics and regulatory mechanisms are as follows:

**(1) Characteristics of Cu speciation transformation**

**Figure 2:**
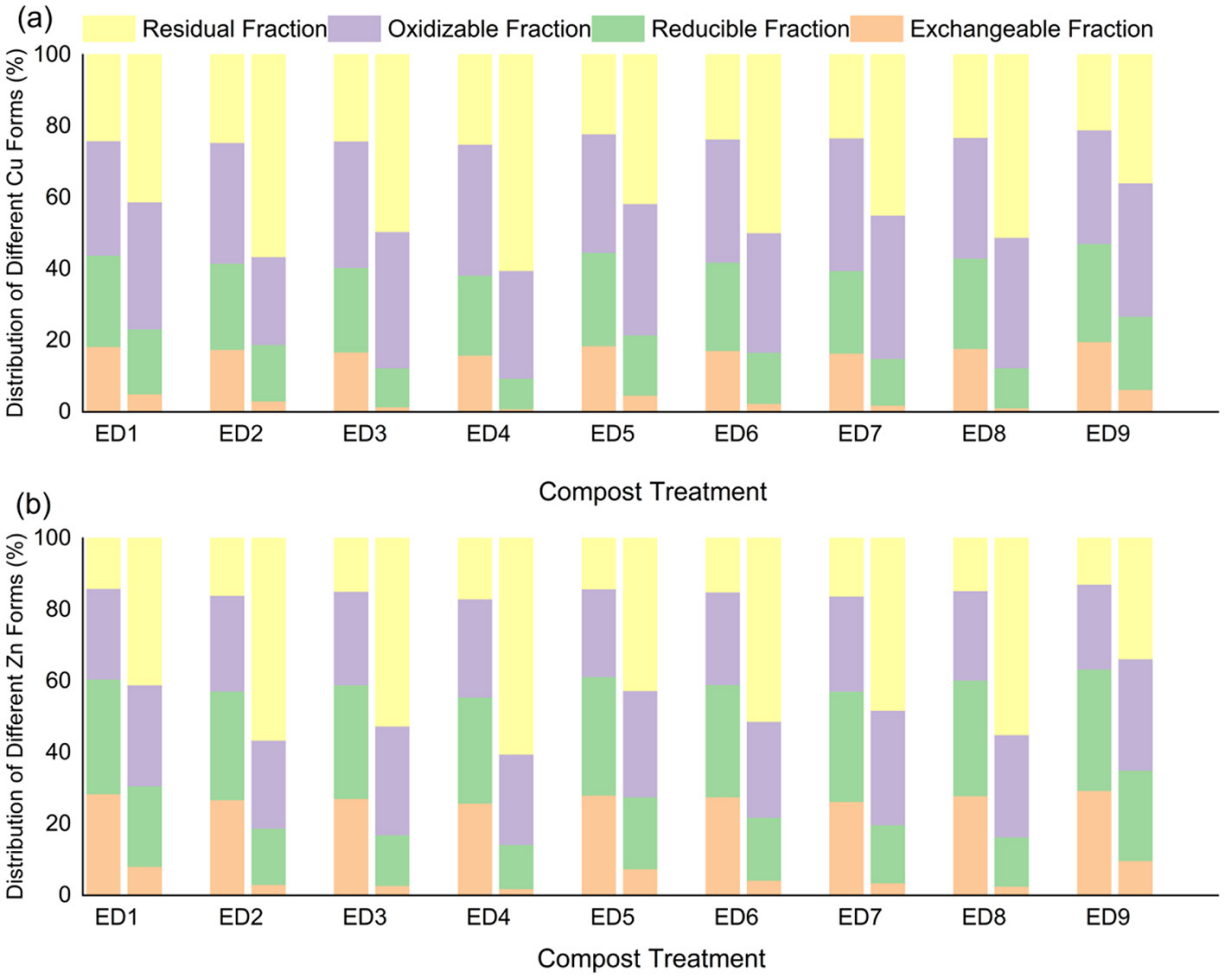
Changes in heavy metal forms in each treatment group (ED1-ED9) during the first and thirty days of composting in the orthogonal experiment: **(a)** Cu; **(b)** Zn.

As shown in Figure 2a, the initial Cu was predominantly in oxidizable (31.8–37.2%) and reducible (22.4–27.5%) states, with the exchangeable state accounting for 15.9–19.7%. After 30 days of composting, the proportions of exchangeable and reducible states significantly decreased in all groups, whereas the proportion of stable states increased. ED4 (60% moisture content, 25:1 C/N, 10% activated carbon) exhibited the best passivation effect, with the residual state increasing from 25.0% to 60.4% and the exchangeable state decreasing to 0.9%. ED9 (60% moisture content, 30:1 C/N, 0% activated carbon) performed the worst, with the residual state being only 35.8%. Activated carbon fixes Cu^2+^ through adsorption and complexation, and C/N = 25:1 promotes humic acid synthesis (chelating with Cu^2+^), jointly enhancing passivation efficiency (Karlsson and Skyllberg, 2007). This synergistic mechanism, which leverages both organic and inorganic amendments, aligns with established principles for heavy metal immobilization during composting as reviewed by Zhong et al. (2024).

**(2) Characteristics of Zn speciation transformation**

Zn has a higher initial activity than Cu (exchangeable state 25.9–29.4%, residual state 12.8–16.9%), but its transformation pattern was consistent with that of Cu, and its overall passivation efficiency was slightly lower (Figure 2b). After 30 days of composting, ED4 still performed the best, with the residual state increasing from 16.9% to 60.4%, and the exchangeable state decreasing to 1.9%; ED9 performed the worst, with the residual state being only 33.7%. Zn is more sensitive to moisture content. In the high moisture content group (65%), due to hindered ion diffusion, the passivation effect is lower than that in the 55–60% group; and Zn has a weaker complexation ability with organic matter than Cu (complexation constant log K = 8.2 < Cu 10.5), resulting in a generally lower proportion of stable state than Cu. Ramaswamy et al. (2010). further confirmed through comparative experiments on pig manure composting that humic acid only increased the stable state of Zn by 22.7% under high moisture content (65–70%), whereas sawdust charcoal in the 55–60% moisture content group increased the stable state to 27.6%. Zhuang (2023) also found that at the end of composting, the exchangeable state proportion of Zn (38.23%) was much higher than that of Cu (11.48%), and the stable state proportion was always 8–12% lower than that of Cu. This difference is due to Zn being more prone to combining with aliphatic compounds in humic acid, with weaker complexation stability, making it difficult to form a durable and stable chelate.

**(3) Range analysis and variance analysis**

The range and variance analysis of the residual proportions of Cu and Zn over 30 days (Tables 3, 4) revealed that the primary and secondary influencing factors were in the order of activated carbon addition (C) > C/N (B) > moisture content (A): activated carbon addition had the largest range (Cu: R = 17.6, Zn: R = 17.1) and the impact reached a highly significant level (p < 0.01); C/N followed closely (Cu: R = 8.5, Zn: R = 9.2), with the impact reaching a significant level (p < 0.05); moisture content had the least impact (Cu: R = 3.9, Zn: R = 1.8), with significant effects only on Cu (p < 0.05). The optimal level was A_2_B_2_C_3_ (60% moisture content, 25:1 C/N, 10% activated carbon), consistent with the ED4 treatment group, confirming that this combination synergistically enhances heavy metal passivation through “activated carbon adsorption-humic acid complexation-maintenance of aerobic environment”, which is consistent with the research conclusion of Zhang et al. (2025) on “enhanced heavy metal passivation by combining microorganisms and biochar”.

**Table 3:** Range analysis for the passivation rate of heavy metals (Cu, Zn) in orthogonal experiments.

**Evaluation metric**	**Factor**	**Level 1(K_1_)**	**Level 2(K_2_)**	**Level 3(K3)**	**Range (R)**	**Primary and secondary order**	**Optimal level**
Cu passivation rate (%)	Water content (A)	30.7	37.6	33.9	6.9	3	A2
	C/N(B)	33.9	44.4	39.9	10.5	2	B2
	Activated charcoal (C)	25.5	38.7	51.3	25.8	1	C3
Zn passivation rate (%)	Water content (A)	39.5	45.3	40.7	5.8	3	A2
	C/N (B)	40.7	51.9	43.5	11.2	2	B2
	Activated charcoal (C)	32.1	45.8	59.4	27.3	1	C3

**Table 4:** Variance analysis of orthogonal experiments on passivation rate of heavy metals (Cu, Zn).

**Evaluation metric**	**Factor**	**Sum of squares**	**Degrees of freedom**	**Mean square**	***F*-value**	***P*-value**	**Significance**
Copper passivation rate (%)	Water content (A)	143.2	2	71.6	9.8	0.019	^*^
	C/N (B)	330.5	2	165.3	22.6	0.002	^**^
	Activated charcoal (C)	1,996.8	2	998.4	136.6	<0.001	^***^
	Error	36.5	5	7.3	-	-	-
Zn passivation rate (%)	Water content (A)	101.2	2	50.6	4.3	0.0073	n.s.
	C/N (B)	376.3	2	188.2	16.1	0.006	^**^
	Activated charcoal (C)	2,237.1	2	1,118.6	95.7	<0.0001	^**^
	Error	58.4	5	11.7	-	-	-

Significance codes: ^***^p < 0.001, ^**^p < 0.01, ^*^p < 0.05, *n.s*. (not significant).

#### 3.1.3 The impact of orthogonal experiments on antibiotic degradation

All three tetracycline antibiotics (TC, OTC, CTC) undergo continuous degradation during composting, with the degradation rates being in the order of OTC > TC > CTC (related to molecular stability). The key characteristics and regulatory mechanisms are as follows:

**(1) Degradation kinetics of tetracycline antibiotics**

**Figure 3:**
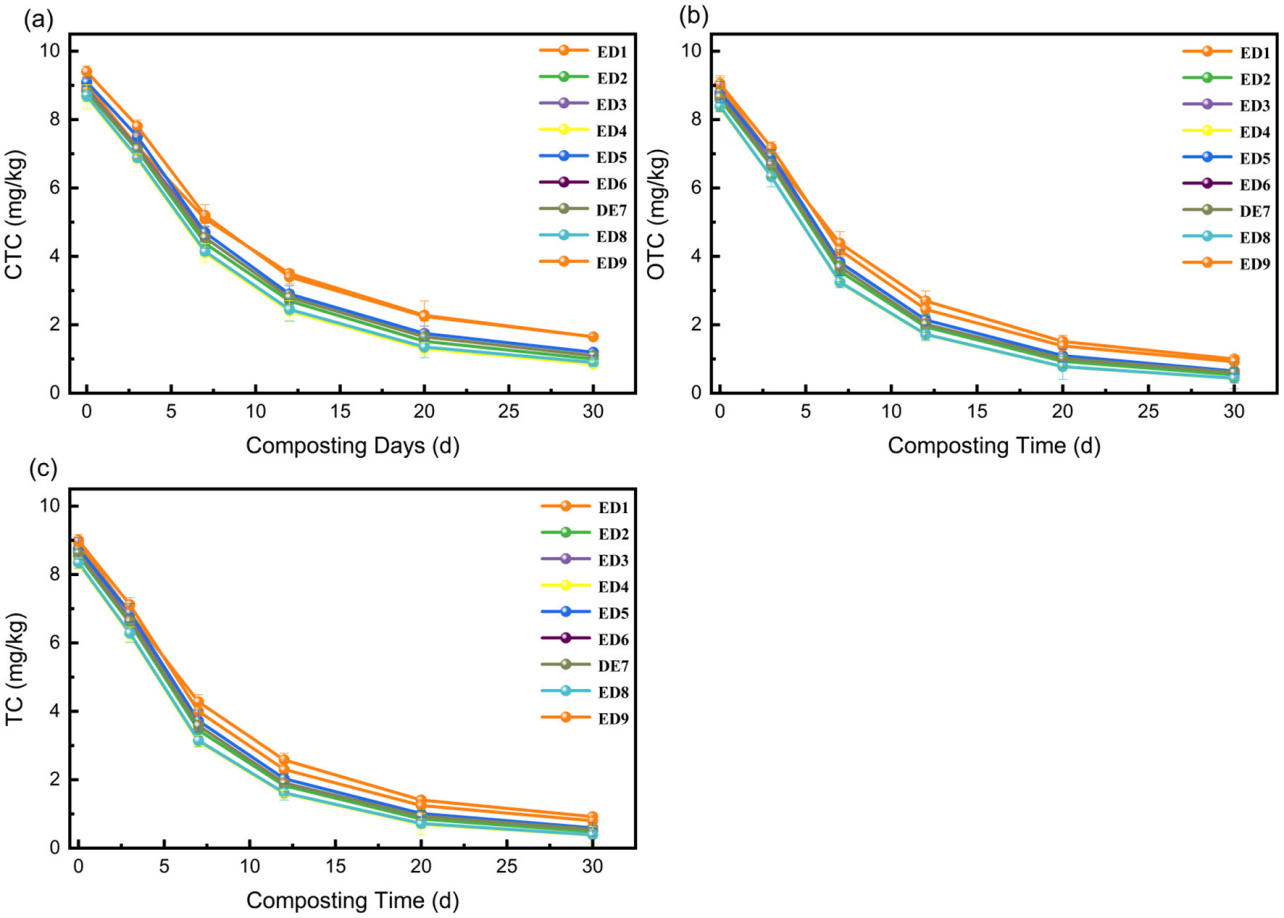
Changes in antibiotic residues in compost from each treatment group (ED1-ED9) in the orthogonal experiment: **(a)** CTC; **(b)** OTC; **(c)** TC.

As shown in Figure 3, the initial antibiotic residues ranged from 8.32 to 9.40 mg/L, which were significantly reduced after 30 d of composting, with degradation rates generally exceeding 85%. ED4 (60% moisture content, 25:1 C/N, 10% activated carbon) performed the best, with TC, OTC, and CTC residues reaching 0.38 mg/L (degradation rate 96.2%), 0.43 mg/L (94.9%), and 0.85 mg/L (90.2%), respectively. ED9 (60% moisture content, 30:1 C/N, 0% activated carbon) performed the worst, with residues of 0.80 mg/L (91.1%), 0.92 mg/L (89.8%), and 1.65 mg/L (82.4%), respectively. TC and OTC degrade rapidly due to their molecules being easily hydrolyzed by enzymes (amide bond and hydroxyl group breakage), while CTC degrades slowly due to the stability of its chlorine atom (C-Cl bond), requiring a synergistic effect of activated carbon adsorption and microbial action (Yamamoto et al., 2010).

**(2) Range analysis and variance analysis**

The range and variance analysis of antibiotic residues over 30 days (Tables 5, 6) revealed that the primary and secondary influencing factors were in the order of activated carbon addition (C) > C/N (B) > moisture content (A); activated carbon addition had the largest range (CTC: R = 0.47, OTC: R = 0.30, TC: R = 0.26), with a highly significant impact (p < 0.01), followed closely by C/N closely (CTC: R = 0.35, OTC: R = 0.28, TC: R = 0.25), with a significant impact (p < 0.05). Moisture content had the least impact (CTC: R = 0.18, OTC: R = 0.15, TC: R = 0.12), with no significant effect (*p* > 0.05). The optimal level was A_2_B_2_C_3_ (60% moisture content, 25:1 C/N, 10% activated carbon), which was consistent with ED4. Its high degradation efficiency stems from “activated carbon adsorption and enrichment, maintaining enzyme activity with suitable parameters, and synergistic multiple degradation pathways”, aligning with Cheng, Liu, Shehata, Feng, Lin and Xue (2021) research conclusion on “functional microorganisms and environmental parameters synergistically enhancing antibiotic degradation”.

**Table 5:** Range analysis of antibiotic residue orthogonal experiment.

**Evaluation metric**	**Factor**	**Level 1(K_1_)**	**Level 2(K_2_)**	**Level 3(K3)**	**Range (R)**	**Primary and secondary order**	**Optimal level**
CTC residual amount	Water content (A)	0.72	0.85	0.93	0.18	3	A1
	C/N(B)	1.08	0.85	1.00	0.35	2	B2
	Activated charcoal (C)	1.28	0.98	0.81	0.47	1	C3
OTC residual amount	Water content (A)	0.58	0.63	0.74	0.15	3	A1
	C/N(B)	0.82	0.54	0.68	0.21	2	B2
	Activated charcoal (C)	0.82	0.58	0.43	0.30	1	C3
TC residual amount	Water content (A)	0.56	0.62	0.73	0.12	3	A1
	C/N(B)	0.77	0.48	0.66	0.25	2	B2
	Activated charcoal (C)	0.77	0.54	0.39	0.26	1	C3

**Table 6:** Variance analysis of antibiotic residue orthogonal experiment.

**Evaluation metric**	**Factor**	**Sum of squares**	**Degrees of freedom**	**Mean square**	***F*-value**	***P*-value**	**Significance**
CTC Residual amount	Water content (A)	0.04	2	0.02	2.8	0.135	n.s.
	C/N (B)	0.15	2	0.075	10.7	0.013	^*^
	Activated charcoal (C)	0.32	2	0.16	22.9	0.002	^**^
	Error	0.035	5	0.007	-	-	-
OTC residual amount	Water content (A)	0.03	2	0.015	2.1	0.208	n.s.
	C/N (B)	0.12	2	0.06	8.6	0.027	^*^
	Activated charcoal (C)	0.21	2	0.105	15.0	0.006	^**^
	Error	0.035	5	0.01	-	-	-
TC residual amount	Water content (A)	0.02	2	0.01	1.4	0.312	n.s.
	C/N (B)	0.10	2	0.05	7.1	0.041	^*^
	Activated charcoal (C)	0.18	2	0.09	12.9	0.008	^**^
	Error	0.035	5	0.007	-	-	-

Significance codes: ^***^p < 0.001, ^**^p < 0.01, ^*^p < 0.05, *n.s*. (not significant).

#### 3.1.4 Comprehensive optimization and parameter determination

Based on the comprehensive objectives of “physicochemical stability (pH 7.0–8.5, EC < 1,000 μS/cm, moisture content 20–30%, organic matter degradation rate > 40%), heavy metal passivation (Cu/Zn residual state > 50%), and antibiotic degradation (degradation rate > 90%)”, combined with the results of range and variance analysis, the optimal parameter combination was determined to be A_2_B_2_C_3_ (moisture content 60%, C/N 25:1, activated carbon addition 10%), corresponding to the ED4 treatment group.

The indicators of ED4 group are optimal: its physicochemical properties meet the composting standards, with a heavy metal residual rate of 60.4% and an antibiotic degradation rate of > 90%. Its synergistic mechanism involves “maintaining an aerobic environment with a moisture content of 60%, achieving balanced nutrition with a C/N of 25:1, and utilizing 10% activated carbon for adsorption, fixation, and bacterial enrichment”. The amount of activated carbon added to this combination (10%) was slightly higher than the conventional amount (5–8%). Owing to the high initial concentration of pollutants in this experiment, a higher dosage is required to enhance the effect, providing a technical reference for the treatment of livestock and poultry manure with a high pollution load.

In terms of physicochemical properties, the optimal combination (60% moisture content, 25:1 C/N, 10% activated carbon) can maintain the stability of the composting environment and meet the maturity criteria. In terms of heavy metal passivation, this combination achieved a residual state of Cu and Zn of 60.4%, with activated carbon being the core regulating factor (*p* < 0.01). In terms of antibiotic degradation, this combination achieved a degradation rate of > 90% for TC, OTC, and CTC, with activated carbon and C/N synergistically enhancing the efficiency. The optimal parameters determined were a moisture content of 60%, C/N of 25:1, and activated carbon addition of 10%, which can provide a basis for the harmless treatment of highly polluted livestock manure.

### 3.2 Ultra-high temperature composting verification experiment

#### 3.2.1 Changes in composting temperature

**Figure 4:**
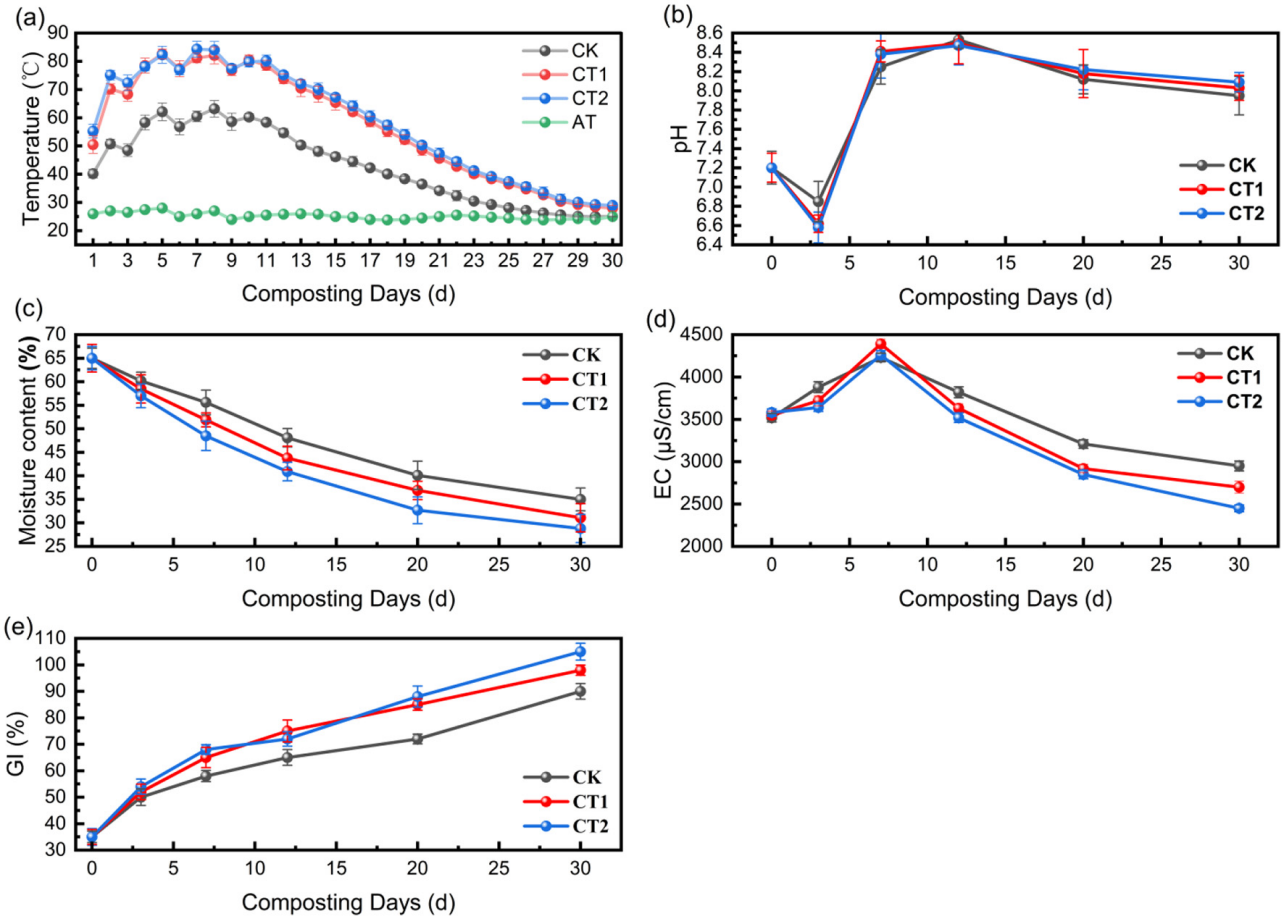
Physicochemical properties of three treatment groups (CK, CT1, CT2) in ultra-high temperature composting: **(a)** Temperature; **(b)** pH; **(c)** Moisture content; **(d)** EC; **(e)** GI.

Temperature is a core indicator reflecting the metabolic intensity of microorganisms and the decomposition efficiency of organic matter. The dynamic temperature changes in the three treatment groups exhibited significant differences from the ambient temperature (23.8–28 °C). The microbial inoculum group demonstrated a superior rate of temperature increase and the ability to maintain ultra-high temperatures (≥70 °C) (Figure 4a).

The control group (CK) achieved a basic temperature increase based on orthogonal optimal parameters. However, limited by the heat tolerance of indigenous microorganisms, the maximum temperature reached only 62.1 °C, never exceeding the ultra-high temperature threshold of 70 °C. The duration of the medium-to-high temperature range (50–63 °C) was only 12 days, making it difficult to rapidly decompose refractory organic matter such as cellulose and hemicellulose. The low-dose microbial inoculum group (CT1) showed a significant improvement in temperature rise efficiency due to the introduction of exogenous microbial inoculum. It reached 70.2 °C (the starting point of ultra-high temperature) on the second day, with a maximum temperature of 82.6 °C, and sustained ultra-high temperature for 14 days, an extension of 2 days compared to the CK group. The high-dose microbial inoculum group (CT2) performed the best, reaching a temperature of 55.3 °C on the first day (a 37.6% increase compared to the CK group's 40.2 °C at the same time), exceeding 70 °C on the second day (reaching 75.1 °C), with a maximum temperature of 84.3 °C throughout the entire cycle, and sustained ultra-high temperature for 15 days.

This difference can be attributed to the metabolic characteristics of the UHT bacterial inoculum: the initial bacterial population density in the CT2 group reached 10^7^ CFU/g (twice that of the CT1 group), proliferating rapidly in a medium-to-high temperature environment, with the bacterial count surging to 10^9^ CFU/g within 1–5 days, exhibiting high heat production efficiency; moreover, the thermotolerant bacterial strains can maintain their activity at 70–85 °C, preventing the inactivation of indigenous bacteria at high temperatures, resulting in a 40–50% increase in the degradation rate of organic matter compared to the CK group (Raave et al., 2014).

#### 3.2.2 Dynamic changes in pH

The pH affects the composting process by regulating the structure of microbial communities and enzyme activities (such as cellulase and protease). All three treatments exhibited a typical composting pattern of “initial decrease, subsequent increase, and final stabilization”, and the pH dynamics in the microbial inoculum group were more aligned with the requirements for compost maturity (Figure 4b).

The initial pH values of all three groups stabilized at 7.2 due to the orthogonal optimal parameter configuration (with the same raw material ratio and 10% activated carbon addition) of the initial compost pile. The alkaline substances (ammoniacal nitrogen) in livestock manure and acidic substances (organic acids) in straw reached a balance, and the low addition of microbial inoculum (0.5–1%) did not significantly disturb the acid-base environment. As composting progressed, the pH values of all three groups showed a downward trend, with a greater decrease in the microbial inoculum group: the CK group decreased to 6.85, the CT1 group decreased to 6.62, and the CT2 group decreased to 6.58. This was due to the secretion of small organic acids such as acetic acid and lactic acid during the initial proliferation phase of the UHT microbial inoculum (the organic acid content in the CT2 group was 120–150 mg/kg, higher than the 80 mg/kg in the CK group). Subsequently, the rate of organic nitrogen mineralization to produce ammonia (NH_3_, ${\text{NH}}_{4}^{+}$) exceeded the rate of organic acid accumulation, and the pH values of all three groups gradually rebounded, but the peak values in the microbial inoculum group were slightly lower (8.49 in the CT1 group, 8.47 in the CT2 group, and 8.53 in the CK group). This was because the UHT microbial inoculum could initiate nitrification in advance, converting some ammonia nitrogen into nitrate nitrogen (${\text{NO}}_{3}^{-}$) and reducing ammonia volatilization losses. By the end of 30 days of composting, the pH values of all three groups stabilized at 7.95–8.09, meeting the “pH 7.0–8.5” standard of “Organic Fertilizers” (GB/T 25246-2010). Among them, the pH value of the CT2 group was closest to neutral-alkaline, which could avoid disturbing the soil acid-base balance after returning to the field.

#### 3.2.3 Changes in moisture content

The moisture content directly affects the aeration of the compost pile, efficiency of material diffusion, and metabolic activity of microorganisms. The moisture content of all three treatments continued to decrease as composting progressed, and the final moisture content of the inoculum group was closer to the ideal range for compost maturity (20–30%) (Figure 4c).

The initial moisture content of all three groups was set at 60%, based on two considerations: firstly, to offset the rapid water evaporation in ultra-high temperature environments, preventing premature drying of the pile that could limit microbial metabolism; secondly, to complement the porous structure (specific surface area of 800–1,200 m^2^/g) of 10% activated carbon, by forming a dynamic equilibrium of “adsorption-release” through the adsorption of free water by activated carbon, thereby assisting in maintaining stable moisture content in the pile (Wang et al., 2022).

During the composting process, the rate of moisture content reduction followed the pattern of “CT2 > CT1 > CK”. Owing to the ultra-high temperature environment (70–84 °C), the moisture evaporation rate in the CT2 group reached 1.2–1.5%/d, which is twice that of the CK group. By the end of 30 days, the moisture content in the CK group decreased to 35% (slightly higher than the ideal range, which is prone to local anaerobiosis), that in the CT1 group decreased to 31.1% (close to the ideal range), and that in the CT2 group decreased to 28.8% (completely within the ideal composting range of 20–30%). Moreover, the appropriate moisture content maintains the porosity of the compost pile at 35–40%, providing sufficient oxygen for microbial metabolism (Ajmal et al., 2021).

#### 3.2.4 Changes in EC

The EC value reflects the concentration of soluble ions (such as NH4^+^, K^+^, Cl^−^, PO_4_^3−^) in the compost pile. These dynamic changes indicate a balance between the release of ions from organic matter mineralization and the absorption and fixation of ions by microorganisms. This is a key indicator for evaluating the salt safety of compost products (Figure 4d).

The initial EC values of the three groups showed minimal differences: CK group at 3,520 μS/cm, CT1 group at 3,550 μS/cm, and CT2 group at 3,580 μS/cm. These slight variations were attributed to the presence of a small amount of soluble salt in the microbial inoculum carrier. However, owing to the low addition of the microbial inoculum, the initial salt level was not significantly affected. As composting progressed, the rate of organic matter mineralization releasing ions exceeded the rate of microbial absorption, resulting in peak EC values for all three groups (CK group at 4,230 μS/cm, CT1 group at 4,390 μS/cm, and CT2 group at 4,260 μS/cm).

Subsequently, the microorganisms continuously absorbed ions to synthesize biomass, whereas activated carbon adsorbed and fixed ions, leading to a gradual decrease in EC in all three groups. By the end of 30 days, the EC of the CT2 group had dropped to 2,450 μS/cm, which was 16.9% lower than that of the CK group (2,950 μS/cm) and 9.3% lower than that of the CT1 group (2,700 μS/cm). This was attributed to the dual regulation of the high-dose microbial inoculum: on the one hand, the high-concentration microorganisms (10^9^ CFU/g) in the CT2 group absorbed a large amount of ions for biomass synthesis; on the other hand, the extracellular polymeric substances (EPS) secreted by the microbial inoculum adsorbed ions through hydrogen bonding and electrostatic interactions, reducing free salt content. Furthermore, the final EC value of the CT2 group was below the plant toxicity threshold of 3,000 μS/cm, meeting the standards of the “Limits of Toxic and Harmful Substances in Fertilizers” (GB 38400).

#### 3.2.5 Changes in GI

The GI serves as a direct indicator for evaluating the maturity and biological safety of compost, providing a visual representation of the reduction of inhibitory substances (such as organic acids and phenolic compounds) and the accumulation in growth-promoting substances within the compost pile. The GIs of all three treatments significantly increased as composting progressed, with the microbial inoculum group demonstrating superior results (Figure 4d).

The initial GI in all three groups was all 35%. Due to the presence of a small amount of inhibitory substances, such as organic acids and phenols, in the composting materials, there was an adverse effect on the germination of Chinese cabbage seeds. During the composting process, the microbial inoculum group experienced accelerated degradation of inhibitory substances because of the UHT environment. At the same time, the microbial inoculum metabolized and secreted enzymes to decompose toxic substances, resulting in a significantly higher germination rate than that of the CK group. The CT2 group also produced growth-promoting substances such as free amino acids (500–600 mg/kg) and humic acid (15–20 g/kg), further promoting seed germination.

By the end of 30 days, the GI of the CK group reached 90% (basically decomposed), that of CT1 group reached 98% (completely decomposed), and that of the CT2 group reached 105% (exceeding 100% due to the effect of growth-promoting substances). All met the decomposition requirement of “GI ≥ 85%” specified in “Organic Fertilizers” (GB/T 25246-2010), and the CT2 group had a significant advantage in fertilizer efficiency.

### 3.3 Enhancing effect of ultra-high temperature on the stabilization of heavy metal forms

**Figure 5:**
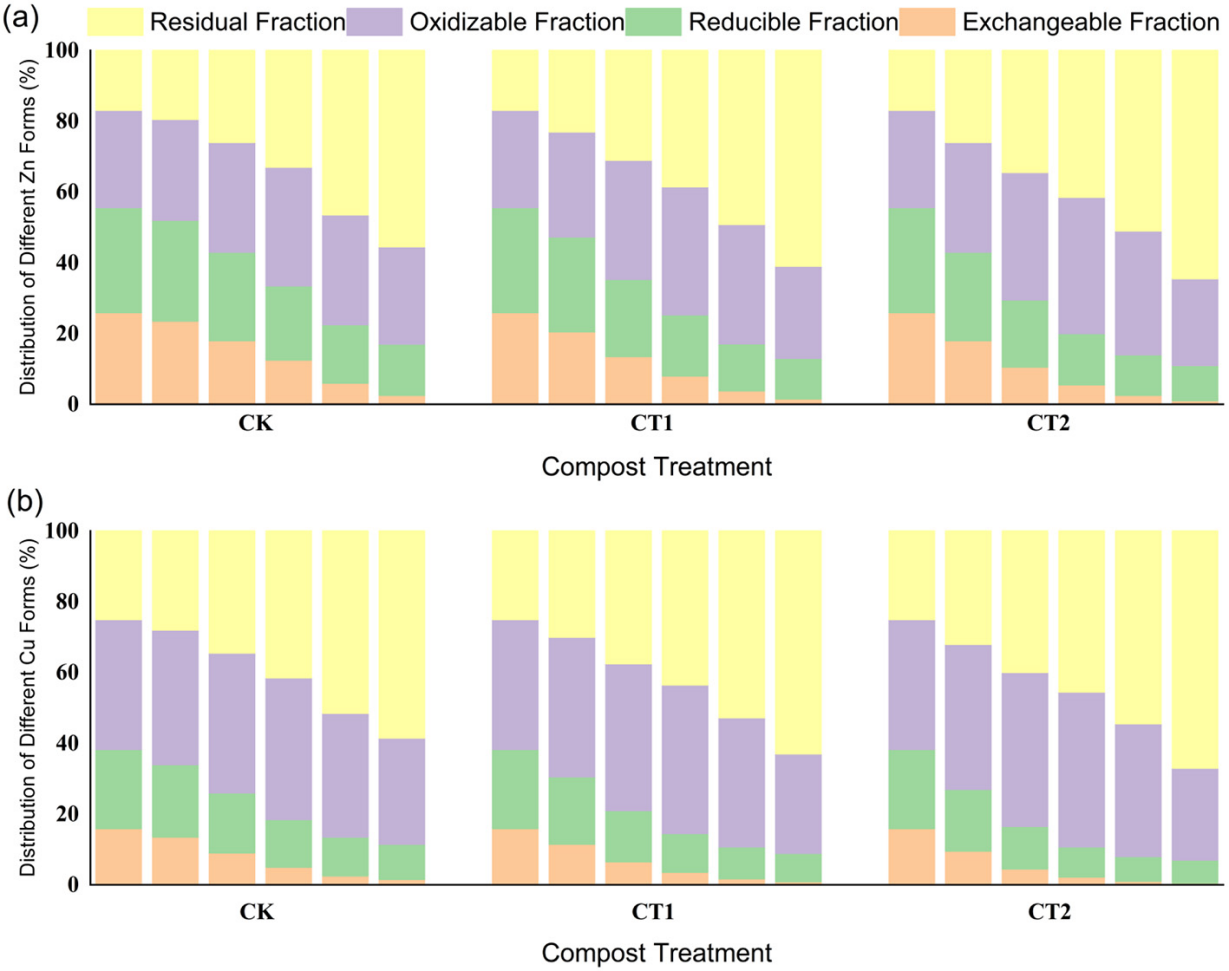
Changes in heavy metal forms in three treatment groups (CK, CT1, CT2) during ultra-high temperature composting: **(a)** Zn; **(b)** Cu.

The bioavailability of heavy metals is directly determined by their morphological distribution. Among them, the acid extractable fraction (a high-risk, easily bioavailable form) decreases stepwise with increasing ultra-high temperature treatment intensity (CT2 > CT1 > CK) and extending composting period (Figure 5). It is clearly shown that the morphological change trends in different treatment groups are highly consistent: taking Cu as an example, at 30 d of composting, the proportion of acid extractable fraction in the CK group was 1.5%, which dropped to 0.9% in the CT1 group and further to 0.5% in the CT2 group; the proportion of acid extractable fraction of Zn also decreased from 2.5% in the CK group to 1.5% in the CT1 group and 1.0% in the CT2 group, with the acid extractable fraction in the CT2 group consistently being at the lowest level throughout the process, visually confirming the inhibitory effect of ultra-high temperature on high-risk forms (Zhou et al., 2018).

As a stable and low-risk form, the residual state (lattice-bound state) exhibited a significant upward trend with increasing ultra-high temperature treatment intensity (Figure 5). At 30 days, the proportion of Cu in the residual state increased from 58.5% in the CK group to 63.0% in the CT1 group and 67.0% in the CT2 group; the proportion of Zn in the residual state increased from 55.5% in the CK group to 61.0% in the CT1 group and 64.5% in the CT2 group. The residual state curve in the figure continued to rise with time, and the slope was steepest in the CT2 group, indicating a stronger stabilization efficiency (Zhou et al., 2018; Zhang et al., 2025). In addition, the oxidizable state exhibits a characteristic of “accumulation in the early stage and transformation in the later stage”. For example, the proportion of oxidizable state in the Cu CK group reaches 40.0% at 12 days and decreases to 30.0% at 30 days; the reducible state (bound to Fe/Mn oxides) continues to decrease. The dynamic changes of these two forms complement each other in the figure, reflecting the gradual transformation of heavy metals from transitional states to stable states under high temperatures (Zhang et al., 2025).

### 3.4 Enhancing effect of ultra-high temperature on antibiotic degradation efficiency

**Figure 6:**
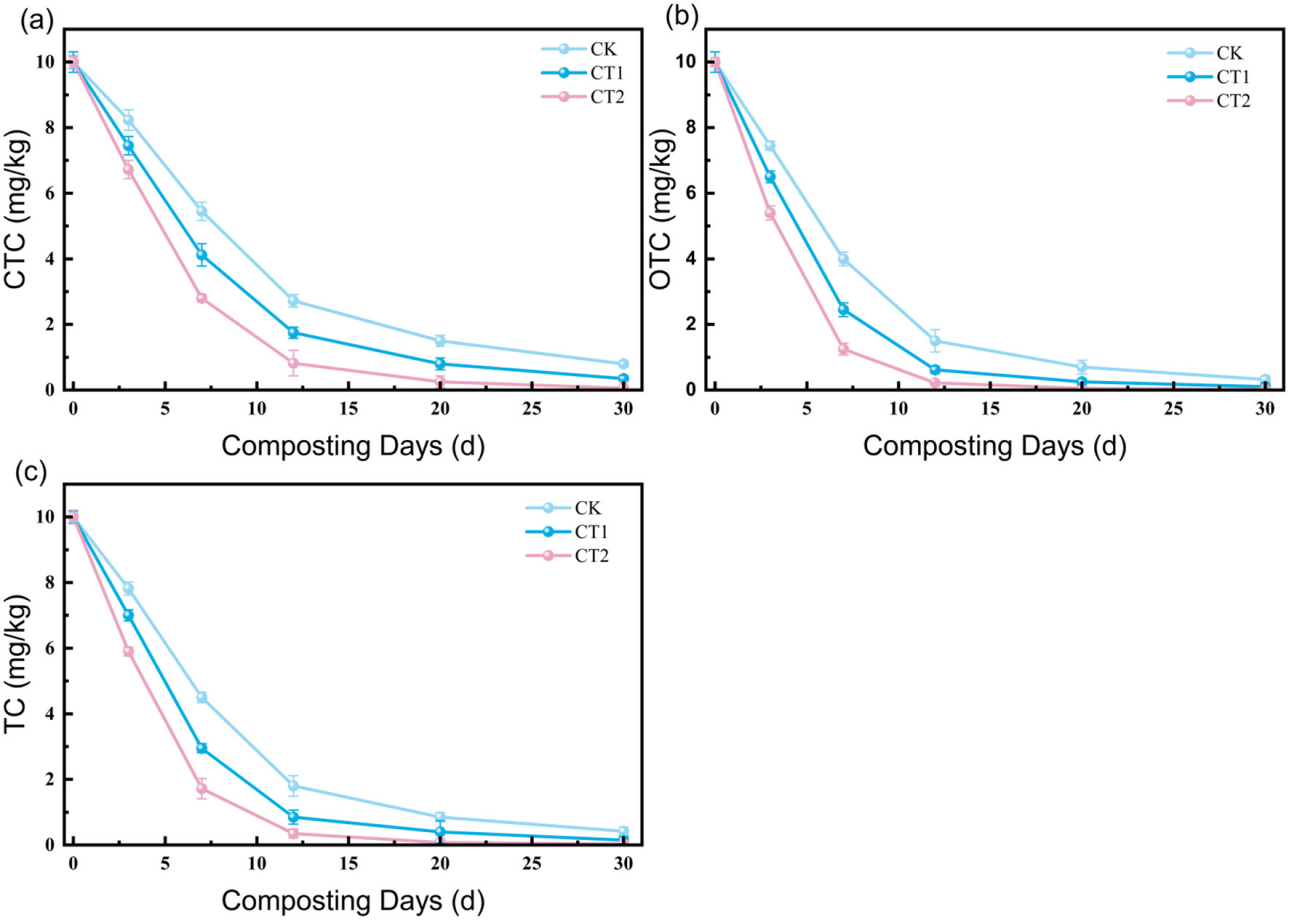
Dynamic changes of antibiotics in three treatment groups (CK, CT1, CT2) of ultra-high temperature composting: **(a)** CTC; **(b)** TC; **(c)** OTC.

The degradation efficiency of TC, OTC, and CTC was significantly enhanced with increasing UHT treatment intensity, exhibiting characteristic rapid early-phase kinetics (Figure 6). The steepest degradation slope in the CT2 group is consistent with a synergistic effect that surpasses a mere thermal contribution. This enhanced efficacy can be attributed to the confluence of two primary pathways supported by the literature and our experimental observations.

#### 3.4.1 Abiotic thermal hydrolysis

The sustained UHT conditions (≥70 °C) directly provide the kinetic energy for accelerated hydrolytic cleavage of labile bonds (e.g., amide bonds) in antibiotic molecules, constituting a fundamental non-biological degradation route. This is corroborated by studies showing that hyperthermophilic composting conditions can significantly accelerate the removal of organic pollutants, including antibiotic resistance genes, through enhanced thermal degradation processes (Liao et al., 2018).

#### 3.4.2 Microbially catalyzed degradation

**Figure 7:**
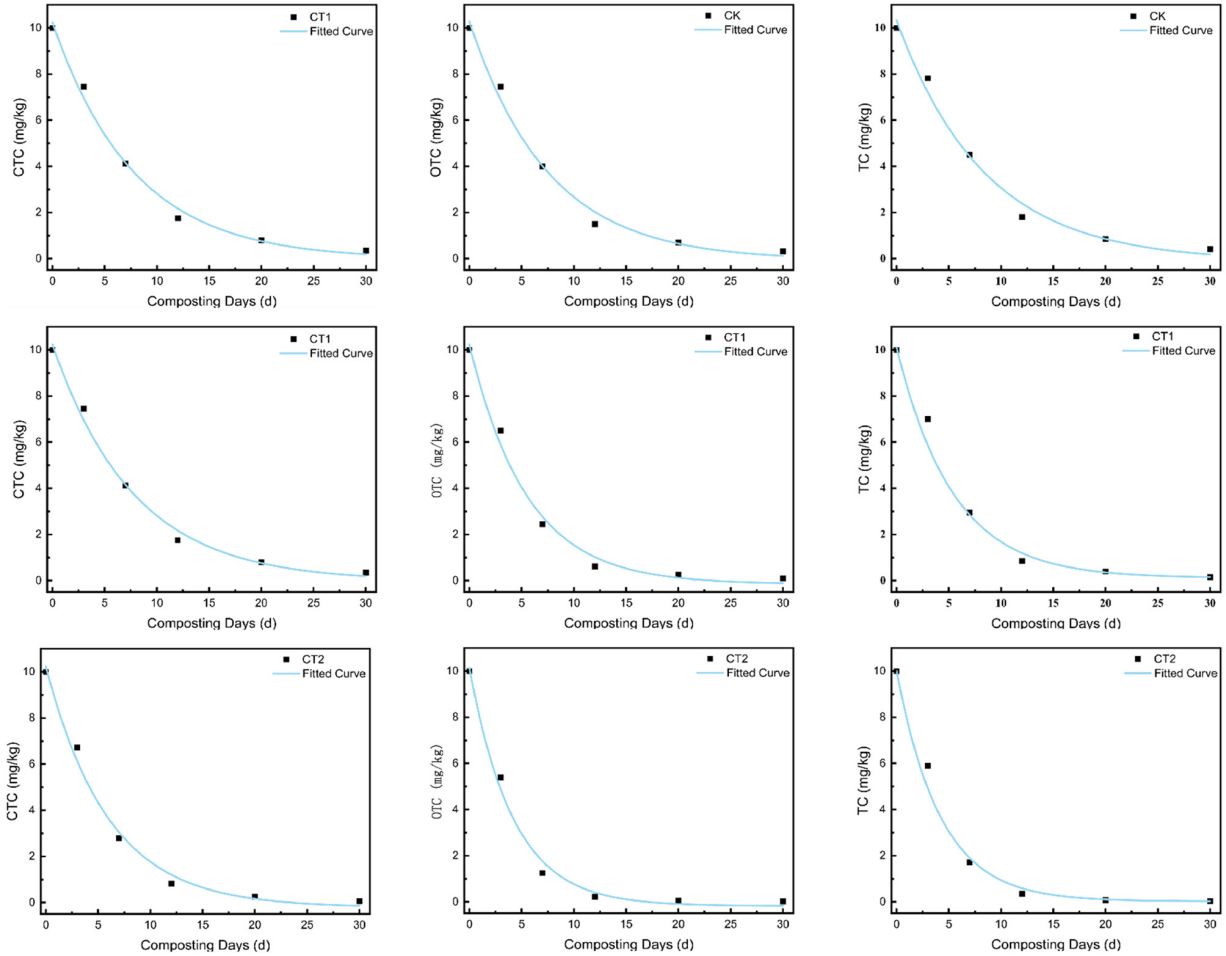
Antibiotic degradation kinetics of three treatment groups (CK, CT1, CT2) in ultra-high temperature composting.

The critical role of the inoculated consortium is strongly suggested by the clear dose-response effect (CT2 > CT1 > CK). Thermophilic genera within the UHTMC (e.g., Geobacillus, Thermomyces) are known to produce robust extracellular enzymes (e.g., laccases, peroxidases) that remain active at elevated temperatures. For instance, research on Geobacillussp. has demonstrated its capacity to produce thermostable laccases capable of degrading complex organic molecules like bisphenol A, providing a direct analog for the enzymatic breakdown of antibiotics observed in our system. These enzymes are capable of catalyzing the oxidative cleavage of complex antibiotic structures. The significantly steeper degradation slope in CT2 during the first 7 days (Figure 7) coincides with the period of peak microbial metabolic heat generation and enzyme activity, lending support to the involvement of this thermophilic enzymatic pathway.

In summary, the superior antibiotic removal in the bioaugmented groups likely results from a synergy between pervasive abiotic hydrolysis and targeted microbial enzymatic attack, both enabled by the UHT environment.

### 3.5 The intrinsic mechanism of synergistic action between heavy metals and antibiotics

The UHT composting system appears to facilitate a synergistic interaction between antibiotic degradation and heavy metal passivation, as evidence by the correlated trends in Figures 5, 6. Based on our data and existing literature, we propose that this interaction operates through two interlocked pathways:

**Pathway 1: Antibiotic Degradation Products Facilitate Metal Passivation**. Antibiotics breakdown generates intermediate products (e.g., small organic acids) containing functional groups (e.g., carboxyl, hydroxyl) that can act as effective chelators. These molecules complex with free heavy metal ions (e.g., Cu^+^, Zn^+^), promoting the transformation of metals from the bioavailable acid-extractable fraction to the less mobile oxidizable fraction-a trend consistent with the early accumulation observed in Figure 5. This concept is supported by studies on the costabilization of heavy metals using waste-derived materials, where organic components synergistically promote stable phase formation (Zhou et al., 2024). These metal-organic complexes may subsequently be integrated into stable humic matrices or mineral lattices, leading to a significant increase in the residual fraction. A process enhancing by optimized amendments like specific grain-size zeolite (Zhang et al., 2025).

**Pathway 2: Metal Passivation Reduce Toxicity and Enhance Microbial Activity**. The effective immobilization of heavy metals, evidenced by the reduced bioavailable fraction in CT2 (Figure 5), alleviates their toxic inhibition on the microbial community. This detoxification creates a more favorable microenvironment, thereby supporting the metabolic activity and functional stability of the antibiotic-degrading microorganisms. This mechanism aligns with stabilization/solidification priciples, where metal encapsulation reduces bioavailability and ecotoxicity mitigating stress on microbial activity (Ma et al., 2020). This positive feedback plausibly explains the sustained high degradation rates observed in Figure 6.

In conclusion, our data support a synergistic model: “UHT-driven antibiotic degradation → generation of metal-chelating ligands → enhanced heavy metal passivation → reduced metal toxicity → further support for microbial degradation activity.” This interlinked process, maintained under UHT conditions, provides a testable hypothesis for the efficient co-remediation achieved.

While this study demonstrates the technical efficacy and proposes a mechanistic framework for the integrated process, future research should include comparative trials with other commercial composting agents to benchmark performance, alongside a detailed techno-economic assessment to evaluate the scalability and cost-effectiveness of this approach for industrial adoption.

## 4 Conclusion

This study demonstrates that UHT composting bioaugmented with a high-dose microbial inoculum (CT2) achieves highly efficient co-remediation of heavy metals and antibiotics in livestock manure. The process significantly promotes the passivation of Cu and Zn, transforming them into stable residual fractions, and achieves near-complete degradation (>99.4%) of TC, OTC and CTC. The key advancement of this work lies in proposing a plausible synergistic mechanism underpinning this efficiency. We hypothesize that the synergy operates through a positive feedback loop: the degradation of antibiotics provides chelating ligands that facilitate metal passivation, while the consequent reduction in metal bioavailability alleviates toxicity and supports the microbial activity responsible for further degradation. This study successfully validates this integrated process at a pilot scale, providing optimized parameters and a testable mechanistic hypothesis for future research.

Future research should employ molecular techniques (e.g., metagenomics, metatranscriptomics) to validate this model, precisely quantify the contribution of specific microbial taxa and functional genes to these pathways, and to identify the full spectrum of degradation intermediates, and to evaluate nitrogen transformation pathways and conservation strategies under UHT conditions to enhance the agronomic value of the final compost product, thereby confirming and refining the mechanistic model.

## Data Availability

The original contributions presented in the study are included in the article/supplementary material, further inquiries can be directed to the corresponding author.
